# Optical parametric amplification of sub-cycle shortwave infrared pulses

**DOI:** 10.1038/s41467-020-17247-9

**Published:** 2020-07-08

**Authors:** Yu-Chieh Lin, Yasuo Nabekawa, Katsumi Midorikawa

**Affiliations:** Attosecond Science Research Team, Extreme Photonics Research Group, RIKEN Center for Advanced Photonics, No. 2-1 Hirosawa, Wako, Saitama 351-0198 Japan

**Keywords:** Ultrafast lasers, Ultrafast photonics, Supercontinuum generation

## Abstract

Few–cycle short–wave infrared (SWIR) pulses are useful tools for research on strong–field physics and nonlinear optics. Here we demonstrate the amplification of sub–cycle pulses in the SWIR region by using a cascaded BBO–based optical parametric amplifier (OPA) chain. By virtue of the tailored wavelength of the pump pulse of 708 nm, we successfully obtained a gain bandwidth of more than one octave for a BBO crystal. The division and synthesis of the spectral components of the pulse in a Mach–Zehnder–type interferometer set in front of the final amplifier enabled us to control the dispersion of each spectral component using an acousto–optic programmable dispersive filter inserted in each arm of the interferometer. As a result, we successfully generated 0.73–optical–cycle pulses at 1.8 *μ*m with a pulse energy of 32 *μ*J.

## Abstract (web summary)

Short-wavelength infrared pulses are important for applications in strong field physics and nonlinear optics. Here the authors show multi-stage optical parametric amplification of sub-cycle SWIR pulses with carrier-envelope phase stability.

## Introduction

The development of laser systems producing a high-intensity single-cycle, or even sub-cycle, pulse has become a worldwide trend in the research fields of ultrashort optics, since it paves the way to numerous advance applications in strong-field physics, such as the generation of isolated attosecond pulses^1^, electron acceleration^2^, and the study of wave-packet dynamics in atoms and molecules^3^. The progress of such high-intensity ultrashort optical pulse sources relies on schemes roughly classified into two categories. One is the post pulse-compression scheme of high-energy short pulses with a duration of 10 or 100 fs order delivered from a conventional amplifier system. The spectral bandwidth of the pulse is extended by self-phase modulation in a gas medium^4^ or one or more thin solid plates^5,6^, then the relatively small amount of dispersion (~100 fs^2^ order) is compensated for by using chirped mirrors.

Hassan and coworkers, for example, have demonstrated a waveform synthesizer based on this technique^7–9^. They adopted a gas-filled hollow-core fiber (HCF) to broaden the spectral width and performed the division and synthesis of multiple spectral bands in the broadened spectrum to compensate for the dispersion in each band, resulting in the generation of sub-cycle pulses spanning from the ultraviolet (UV) to near-infrared (NIR) wavelength region. The main issue with an HCF is the limitation of the pulse energy in the sub-mJ range, which is responsible for the scalability of the length and core diameter of an HCF situated in a laboratory with typical dimensions, even though multi-mJ pulses can be generated^10^ in a laboratory including an exceptionally long space. Shumakova et al.^11^ realized the generation of multi-mJ pulses with a few-cycle pulse duration by using the self-compression technique in a solid plate, although the wavelength range was limited to the mid-infrared around 3–5 μm and the bandwidth was not sufficient to form a single-cycle pulse.

To overcome the energy limitation, the amplification of broadband pulses in gain media should be cited as another scheme. In particular, the optical parametric amplification (OPA) of IR pulses in a nonlinear crystal was adopted as a useful scheme in such high-intensity ultrashort pulse sources, because the gain bandwidth in OPA is generally broad owing to the low dispersions of nonlinear crystals in this wavelength region. The gain bandwidth can be further extended to approximately one octave when the degeneracy condition for the wavelengths of the pump, signal, and idler pulses is satisfied. In fact, Ishii et al. developed a mJ-class optical parametric chirped-pulse amplifier system generating sub-two-cycle pulses by utilizing the broadband phase-matching condition of a nonlinear crystal of BIBO at a degenerate wavelength of 800 nm for the pump pulse^12^. Yin et al.^13^ also built a similar laser system with a pulse energy much higher than 1 mJ at the expense of a 30% increase in pulse duration compared with that reported in ref. ^12^. To extend the gain bandwidth and increase the pulse energy, frequency domain OPA has been proposed and demonstrated to provide two-cycle pulses with an energy more than 10 mJ^14,15^.

In spite of the progress toward shortening the pulse in OPA systems, the shortest pulse duration has nor yet reached the sub-cycle regime owing to the insufficient gain bandwidth in a nonlinear crystal used in an OPA system. One of the promising methods for resolving this issue is an optical wave synthesizer^16–19^ consisting of multiple OPA chains, each of which amplifies few-cycle pulses with a distinct wavelength from the others.

Huang et al. reported the generation of 0.8-cycle pulses with an energy of 15 μJ by utilizing this method^16^. The enormous extension of the gain bandwidth in this method enables the formation of an optical transient whose pulse duration is much shorter than one optical cycle at the cost of having to stabilize a long optical path across an optical table in each OPA system with  ~100 nm precision and to develop multiple pumping laser sources with different wavelengths.

In this paper, we report on sub-cycle pulse amplification in the short-wave IR (SWIR) wavelength region by utilizing an OPA. The key feature of this OPA scheme is the wavelength of the pumping pulse. The increase in gain bandwidth so that it spans from 0.9 to 2.4 μm is due to the fact that the wavelength of the pump pulse is tuned to 708 nm, which is a specific wavelength exhibiting broadband phase-matching in a BBO nonlinear crystal. Another feature is a hybrid scheme of waveform synthesis and an OPA chain with a single-color pump source to control the dispersion of an SWIR spectrum spanning over one octave by using two acousto-optic programmable dispersive filters (AOPDFs), which control shared spectral components on the short side (0.9–1.45 μm) and on the long side (1.45–2.4 μm). This is because an AOPDF usually cannot control the dispersion for the whole range of an over-octave-spanning spectrum. We utilize a Mach–Zehnder-type interferometer (MZI) consisting of a spectral beam splitter and a combiner optimized for the division and synthesis of the two spectral components with low dispersion. Each AOPDF for each spectral component is inserted into each arm of the interferometer. Owing to the over-octave-spanning gain bandwidth of the OPA, we can amplify the pulse energy of the synthesized pulse passing through a common path in the OPA. The length of the beam path in the interferometer is designed to be as short as possible so that the phase jitter between the two spectral components is reduced to 130 mrad under active feedback control.

The temporal profile or dispersion of the amplified pulse is evaluated using a two-dimensional shearing interferometer (2DSI)^20,21^, resulting in a pulse duration of 4.3 fs, which is equivalent to 0.73 optical cycles of the carrier wavelength of 1.8 μm. The carrier envelope phase (CEP) estimated by the shot-to-shot measurement of the spectral fringes of *f*–2*f* interference at the repetition rate of the amplified pulse (200 Hz) exhibits an rms error of 493 mrad, which is reasonable for a low-repetition-rate laser system compared with the CEP error in the 10 Hz laser system reported in ref. ^22^. The pulse energy obtained from the OPA chain is 32 μJ with a pump pulse energy of 1.1 mJ. We expect to improve the energy to the mJ-level range by adding an OPA chain with a pumping source on the order of 10 mJ since we have already had an experience to develope a Ti:sapphire laser system suitable for such a pumping source^23^.

## Results

### System overview

**Fig. 1:**
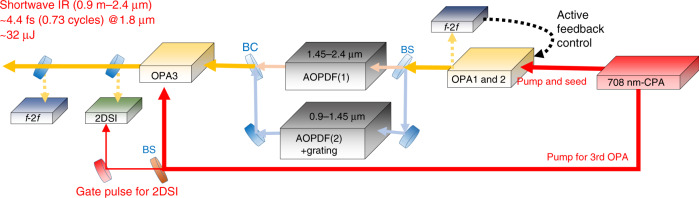
Schematic setup for the OPA system used to amplify sub-cycle SWIR pulses. A laboratory-built Ti:sapphire CPA system delivered 708-nm pulses as the driving source for the three subsequent OPA stages. Between the second and third stages, an MZI was set to divide and synthesize the spectrum for dispersion management with two AOPDFs, one placed in each arm. Part of the pumping energy was used for the pulse characterization (2DSI) system. The relative CEP for the amplified pulses from OPA2 was determined using an *f*–2*f* interferometer and the error signal was fed back to the controller, which adjusted the delay between the pump and seed pulses in OPA1 to stabilize the CEP. 2DSI two-dimensional shearing interferometer, BS beam splitter, BC beam combiner.

**Fig. 2:**
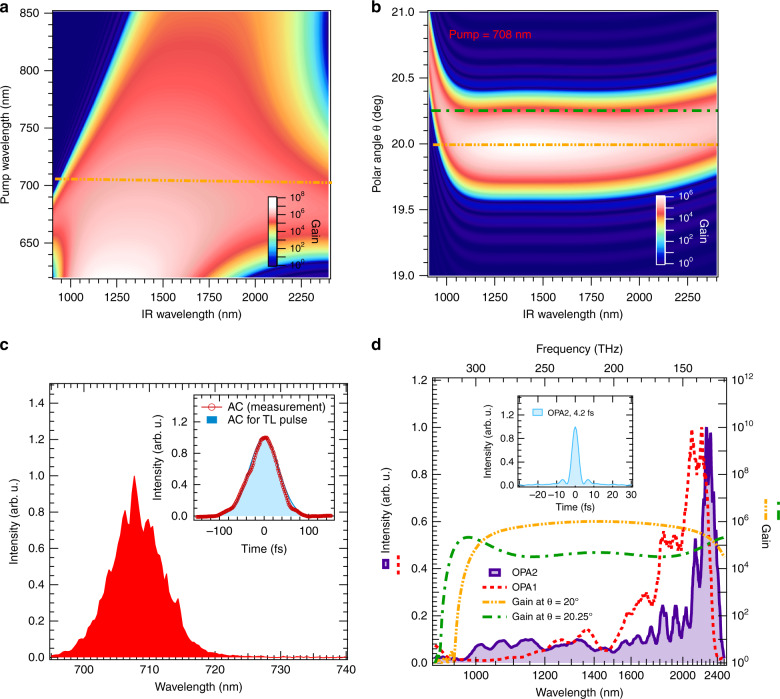
Spectra for the calculated gain of a BBO, pump beam pulse, and output SWIR pulse. **a** Calculated gain spectrum of a 3-mm BBO under the type-I phase-matching condition. The phase-matching angle is adjusted for each wavelength of the pump beam so as to satisfy the degeneracy condition for the signal and idler beams in this calculation. The two-dot chain line indicates the pump wavelength of 708 nm. **b** Calculated gain spectrum at the pump wavelength of 708 nm. Deviation from the complete phase-matching angle *θ* = 20° was scanned to show the change of the profile of the gain spectrum. The two-dot chain curve and the one dot chain curve specify the angle of 20° and 20.25°, respectively. **c** Measured spectrum of the output pulse from the Ti:sapphire CPA system. The autocorrelation (AC) trace measured with a single-shot autocorrelator is shown as circles in the inset. The autocorrelation trace of the transform-limit (TL) pulse calculated from the measured spectrum is also shown as the shaded area in the same inset. **d** Spectra of the output pulses from OPA1 and OPA2 depicted as dashed curve and shaded area, respectively. The calculated gain profile at *θ* = 20° and *θ* = 20.25° with the pumping wavelength of 708 nm are depicted with a two-dot chain, and a one-dot chain curves, respectively. The temporal profile of the TL pulse calculated from the output spectrum of OPA2 is shown in the inset.

The experimental setup is outlined in Fig. 1. It starts with a laboratory-built chirped pulse amplification (CPA) system of a Ti:sapphire laser. The CPA system delivers femtosecond pulses with a wavelength of 708 nm (red) as the driving source for the subsequent three stages of an OPA chain. The dispersion controller accompanying two AOPDFs in an MZI for spectral division and synthesis is set between the second and third OPA stages. Part of the pumping energy is utilized for the 2DSI measurement of the amplified pulse. Another part is also used for the up-conversion of the SWIR to visible pulses so as to find the spectral interference fringes exhibiting the stability of the MZI (not shown in Fig. 1). The spectral profile of the parametric gain as a function of the scanning wavelength of the pumping laser pulse is shown in Fig. 2a. The parametric gain in this figure is simulated for a 3-mm-thick BBO crystal cut for the type-I phase-matching condition at a fixed intensity of the pump under the degeneracy condition for the signal and idler beams. The analytical formulae applied to calculate the parametric gain profile are Eqs. (4)–(6) in “Methods”. We can see from this figure that the gain bandwidth around the 700-nm pump is significantly broader than that around the 800-nm pump and is almost equivalent to one octave, as shown in Fig. 2d (dash-dot-dotted line). This broadening of the gain bandwidth originates from the vanishing second-order dispersion around the 700-nm pump in addition to the vanishing odd-order dispersions under the degeneracy condition for the phase matching as explained in “Methods”. A slight deviation of the cutting angle of the crystal from the complete phase-matching angle causes further broadening of the gain bandwidth to over an octave even though spectral modulation occurs around the center wavelength of the gain profile, as shown in Fig. 2b, which depicts the gain profile as a function of the polar angle *θ* at a fixed pump wavelength of 708 nm. We show the gain profile with a polar angle of 20.25°, which is slightly larger than the complete phase-matching angle of 20°, as a dash-dotted curve in Fig. 2d, as an example of bandwidth broadening. The reason for the bandwidth broadening of the gain profile is discussed in “Methods”.

### Laboratory-built Ti:sapphire CPA system

The configuration of the CPA system of the Ti:sapphire laser was partly described in refs. ^24,25^. In brief, a train of sub-10-fs pulses with a repetition rate of 78 MHz generated from a mode-locked oscillator of a Ti:sapphire laser (Rainbow, Femtolasers-Spectra-Physics) was sent to an Öffner-type stretcher to increase the duration of the pulse to  ~300 ps with the spectral component from 670 to 750 nm restricted by the dimensions of the optics used in the stretcher. A stretched pulse in the chain was amplified in a regenerative amplifier of a Ti:sapphire laser pumped by the second-harmonic pulse of a Nd:YLF laser (Darwin, Quantronix-Continuum) with a repetition rate of 200 Hz and a pulse energy of approximately 5 mJ. We inserted a custom-made dielectric filter (TFMQ-30C02-10-5-TypeswA, Opto-Sigma) in the cavity of the regenerative amplifier to tune the peak gain of the amplifier to  ~700 nm. Note that we can control the wavelength of the amplified pulse by adjusting the incident angle to the dielectric filter. After passing through a pulse slicer consisting of a Pockels cell set between two crossed polarizers, the output pulse was injected to a four-pass amplifier. The pulse energy was increased from 200 μJ to 4.3 mJ in this amplifier and then decreased to 1.9 mJ by passing through a grating pair compressor set behind the four-pass amplifier. We show the measured spectrum of the output pulse from the compressor in the CPA system of the Ti:sapphire laser in Fig. 2c. The peak wavelength and spectral width are approximately 708 and 8 nm in full width at half maximum (FWHM), respectively. The measured autocorrelation trace of the compressed pulse is depicted as circles in the inset of Fig. 2c, which is in good agreement with the autocorrelation trace (solid curve) of the transform-limit (TL) pulse calculated from the measured spectrum. Thus, we conclude that the generated pulse is near the TL with a pulse duration of 60 fs. The energy of the output pulse from the CPA system was measured to be 1.9 mJ. The repetition rate of the output pulse is 200 Hz.

To ensure the pointing stability in the subsequent OPA, we inserted a stabilizing system (Aligna, TEM Messtechnik) for the beam pointing immediately after the compressor.

### First and second OPA stages

The output pulse from the Ti:sapphire CPA system is first split into two paths by a partially reflecting mirror. The reflected pulse is used for the first OPA stage (OPA1) and the transmitted pulse is sent to the subsequent OPA stages and measurement apparatus.

In OPA1, the beam path is further split by a partially reflecting mirror to produce seed and pump pulses. A detailed description of the white-light generation (WLG) used to generate a seed pulse given in “Methods”. The temporal overlap between the seed and pump pulses is also noted in the same subsection in “Methods”. It is well-known that a difference frequency generation (DFG) process in a nonlinear crystal cancels the CEP fluctuation, and thus, we utilize the idler pulse from OPA1 as a seed pulse in the subsequent OPA stages to stabilize the CEP.

We obtained a pulse energy of 9 μJ for the idler pulse with a pump energy of  ~200 μJ and an intensity of  ~60 GW/cm^2^, from a 3-mm-thick BBO crystal cut at *θ* (polar angle) = 20° for the type-I phase-matching condition. The spectrum spans from 0.9 to 2.4 μm, depicted as a dotted curve in Fig. 2d. Note that the spectra of the amplified pulses shown in this figure and the following figures are calibrated in the optical frequency domain indicated on the top axis in each figure. We attempted to enhance the low-frequency part of the spectrum (~140 THz) by adjusting the angle of the optical axis in the BBO crystal and the delay between the pump and seed pulses so that the optical period of the carrier wavelength would be as long as possible. We determine the carrier wavelength from the center-of-gravity (COG) frequency of the measured spectrum in the frequency domain throughout this paper.

In spite of the generation of an over-octave-spanning spectrum, there is an issue to be resolved. We adopted the noncollinear configuration of the pump and seed pulses for the OPA under the type-I phase-matching condition at the degeneracy wavelength to avoid the indistinguishability between the signal and idler pulses. Note that the noncollinear scheme for the pump and seed pulses was applied to all the OPA stages with an angle of less than 1°. This configuration is always accompanied by an angular dispersion of the idler pulse even though we designed the beam paths of the pump and seed pulses to be on the *ϕ* (azimuth angle) plane of the BBO crystal. Therefore, we employed a spatial filter consisting of two concave mirrors and a pinhole. This is because the propagation of the output pulse from the pinhole placed on the image plane of the output surface of the BBO crystal, which is projected by the first concave mirror, is governed by the diffraction from the pinhole rather than the trace of the ray, resulting in the removal of the angular dispersion at the cost of significant reduction of the pulse energy to 0.12 μJ. After the collimation with the second concave mirror, this pulse is sent to the second OPA stage (OPA2).

The spectral components at the shortest and longest edges of the seed pulse injected to the OPA2 are both 100–70 fs delayed to the most advanced wavelength component at around 1.5 μm mainly owing to the third-order dispersion of the BBO crystal in the OPA1. This delay is slightly longer than the pulse duration of the pump pulse in FWHM; thus, we carefully tuned the delay between the seed and pump pulses to transfer the substantial energy component of the pump pulse to near the shortest- and longest-edges of the wavelength components in the seed pulse. As a result, we obtained the output spectrum from OPA2 depicted as a shaded area in Fig. 2d. The spectrum still maintains the over-octave bandwidth, and the resultant pulse duration calculated from this spectrum at the TL is 4.2 fs, corresponding to 0.64 optical cycles of the carrier wavelength of 1.95 μm, as shown in the inset of Fig. 2d. The energy was amplified to 8.6 μJ after the OPA2 with a pump energy of  ~220 μJ and an intensity of  ~60 GW/cm^2^.

### CEP measurement after OPA2

**Fig. 3:**
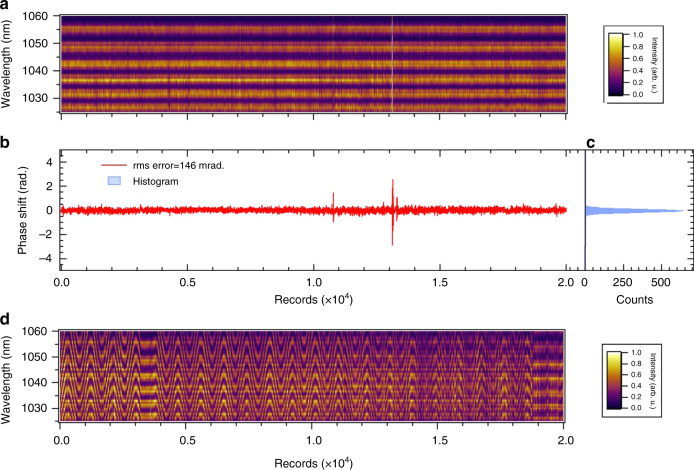
CEP measurement after OPA2. **a** Temporal evolution of the *f*–2*f* spectral interference fringes originating from the output pulse from OPA2 under CEP stabilization control. We performed the three-shot accumulation of the spectrum for each record, and thus, the number of records (2.0 × 10^4^) is equivalent to the recording time of 5 min by considering a pulse repetition period of 5 ms. **b** Relative phase shift (CEP) extracted from the spectrogram depicted in **a**. The rms error of the phase shift is estimated to be 146 mrad. **c** Histogram of the relative phase shift. The width of a bin is 0.01 rad (we set 100 bins/rad) in this histogram. **d** *f*–2*f* spectral interference fringes when we actively modulated the delay between the pump and seed pulses in OPA1 under CEP stabilization control.

We further measured the *f*–2*f* spectral interference fringes of the output pulse from OPA2 to examine the stability of the CEP at this stage. The details of this measurement and the *f*–2*f* interferometer are described in “Methods”. The time evolution of the *f*–2*f* interference fringes is shown in Fig. 3a. This was recorded under the feedback control of a piezo translation stage while adjusting the optical delay between the pump and seed pulses in OPA1 to compensate for the CEP drift in a time range of a few seconds. The resultant rms error of the CEP extracted from this record is estimated to be 146 mrad (in-loop), as shown in Fig. 3b. The histogram of the CEP shift is also shown in Fig. 3c. Note that we can scan the CEP by controlling the phase offset in the feedback loop owing to the DFG scheme for CEP stabilization, as shown in Fig. 3d.

### Dispersion control in an MZI

**Fig. 4:**
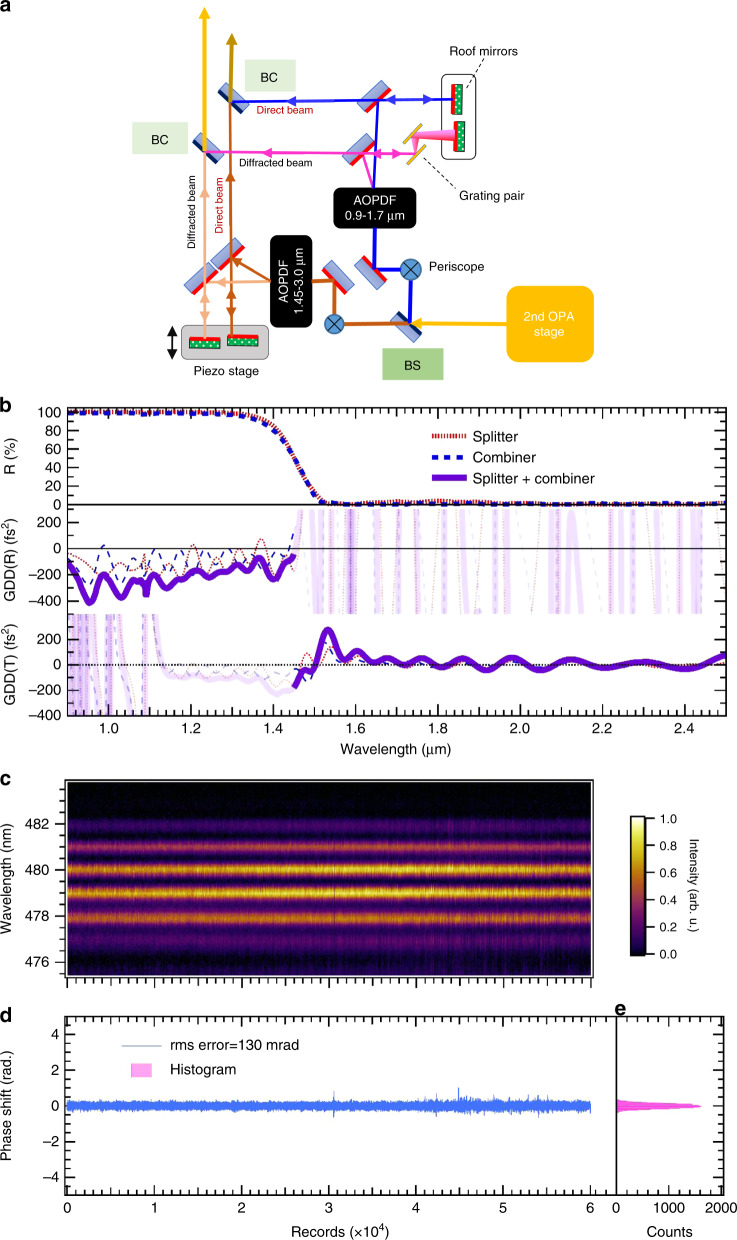
Stabilization of the MZI. **a** Schematic figure of the setup for the dispersion controller using two AOPDFs. The configuration is basically the same as that of the MZI. BS beam splitter, BC beam combiner. **b** Optical properties designed for the BS and BCs. Top panel: the reflectivities designed for the BS and BCs are depicted as dotted and dashed curves, respectively. The wavelength for 50% reflectivity is adjusted to 1450 nm for both the BS and BC. Middle panel: group delay dispersion (GDD) given by a reflection from the BS chirped mirror coating (dotted curve), and that caused by the reflection from the BC (dashed curve). The solid curve represents the total GDD of the BS and BC reflections. The trace color for each GDD in the low-reflectance wavelength region (>1450 nm) is suppressed because only the region of high reflectance is relevant. Bottom panel: GDD given by the transmission of the BS (dotted curve), and that caused by the transmission of the BC (dashed curve). The solid curve represents the total GDD of the BS and BC transmissions. The trace color for each GDD in the low-transmission wavelength region (<1450 nm) is suppressed because only the region of high transmission is relevant. **c** Temporal evolution of the spectral interference fringes between the long- (>1450 nm) and short-wavelength (<1450 nm) components divided and synthesized in the MZI. We utilized the non-diffracted (direct) beams from the AOPDFs for this measurement. The spectral interference fringes appearing in the wavelength region near 1450 nm were up-converted to the visible region by sum-frequency generation with the residual pump pulse from OPA3. The acquisition time for each record of the spectrum is equivalent to the repetition period of the laser pulse (5 ms), and thus, the number of records (6 × 10^4^) represents the recording time of 5 min. **d** Relative phase shift extracted from the spectral interference depicted in **c**. The rms error of the phase shift is estimated to be 130 mrad. **e** Histogram for the relative phase shift. The width of a bin is 0.01 rad (we set 100 bins/rad) in this histogram.

The output pulse from OPA2 is sent to the dispersion controller consisting of two AOPDFs (Fastlite) with different spectral ranges (900–1700 and 1450–3000 nm), one set in each arm of an MZI, as schematically shown in Fig. 4a. This is because the spectral width of the output pulse from OPA2 is broader than the spectral width that can be controlled with one AOPDF. There were two issues when building this interferometer. One was how to split the spectral component into two wavelength regions. The other was the stabilization of the interferometer, which is discussed in the following section (Stabilization of the MZI).

The former issue was resolved by developing broadband chirped dichroic beam splitter and combiner mirrors. The optical properties designed for these mirrors are shown in Fig. 4b. The splitter and combiner mirrors both reflect the wavelength component shorter than 1450 nm, whereas the oscillations of the group delay dispersion (GDD) of the reflected beams from these two mirrors in the short-wavelength region compensate for each other such that the oscillation amplitude should be sufficiently small to avoid the formation of satellite pulses. The GDD oscillations of the pulse transmitted to these two mirrors in the wavelength region longer than 1450 nm are both sufficiently small not to cause a significant temporal modulation of the pulse. Thus, we can neglect the GDD oscillation, which cannot be managed with the AOPDFs, from the splitter and combiner mirrors.

Moreover, the chirped dichroic beam splitter and combiner mirrors were designed to compensate for the deformations of the mirror surfaces with the tension emerging from the accumulated dielectric films of the mirror coatings^8^. The splitter reflects the short-wavelength component on the air side of the mirror coating, while the combiner reflects it on the substrate (made of CaF_2_ with a diameter of 25.4 mm and a thickness of 6 mm) side so that the signs of the wavefront curvature of the reflected beams would be opposite if such surface deformations exist. Furthermore, we fabricated an anti-reflection coating on the surface of another side with a total thickness similar to that of the chirped mirror coatings so as to cancel out the surface tensions of the dielectric films on both surfaces. Hence, the deformations of the surfaces of both the splitter and combiner mirrors are expected to be negligible. The agreement of the wavefronts of the short- and long-wavelength components will be further demonstrated in the Section “Beam focusing and profile”.

By employing the custom beam splitter, the SWIR spectrum from OPA2 was first splitted into two wavelength components and sent to each arm in the MZI. The polarization of each pulse in each arm of the interferometer was rotated 90° from the parallel direction to the perpendicular direction to an optical table surface by a periscope since the polarization of the input beam for the AOPDF should be in the vertical direction. The incident angle to the AOPDFs was carefully tuned with a three-axis stage to maximize the diffraction efficiency. The diffraction efficiencies of the AOPDFs in the short-wavelength and long-wavelength arms can reach to 64% and 65% when optimum dispersions are applied to the AOPDFs so as to maximize the diffraction efficiencies, and they were reduced to 14.3% and 7% under the actual conditions for compensating the dispersion, respectively. The net throughput of the MZI was estimated to be 3%, and thus, the pulse energy at the exit of the MZI is  ~0.2 μJ. The significant reduction in pulse energy in the MZI is mainly due to the low efficiency of the AOPDFs and also the grating pair, which has a net efficiency of  ~40%, inserted in the short-wavelength beam line. More details about the gratings will be described in the following.

In the arm for the short-wavelength component, the diffracted beam under dispersion control with the AOPDF was led by a steering mirror and a roof mirror to a chirped dichroic beam combiner mirror on which the other diffracted beam from the arm for the long-wavelength component was superposed. The optical path length of one arm in the interferometer is reasonable for a conventional interferometer (approximately 0.6 m), resulting in relatively good short-term stability for the relative phase without the active feedback control of the beam path. We inserted a gold-coated reflective grating pair with a groove density of 600 g/mm between the steering mirror and the roof mirror in the arm for the short-wavelength component because it is necessary to precompensate for the large positive chirp around the wavelength of 1 μm originating from the TeO_2_ crystal used in the AOPDF. The separation between the two gratings in the pair was set to approximately 7.5 mm.

In addition, the radio frequencies (RFs) driving the acoustic wave in the AOPDFs were synchronized with the repetition frequency of the mode-locked oscillator in the CPA system to inhibit the CEP jitter originating from the RF phase jitter to the trigger signal for the AOPDFs.

Using the scheme of the MZI with AOPDFs, we successfully solved the difficulty in the dispersion management of the over-octave spectral range.

### Stabilization of the MZI

The stabilization of the relative phase between the short- and long-wavelength components was executed by detecting spectral interference fringes^8^ between the two components near the wavelength edge of the splitter and combiner mirrors (~1450 nm) where the spectra of the reflected and transmitted beams overlap. In the actual setup, however, we cannot use the diffracted beams from the AOPDFs for this purpose since they are temporally overlapped without delay and thus do not exhibit spectral interference fringes. Therefore, we noticed that non-diffracted (direct) beams, which always accompany the diffracted beam from the AOPDF and are usually discarded and terminated, can be used for the purpose.

Each non-diffracted beam output from the AOPDF in each arm passes through a half-wave plate to rotate its polarization from perpendicular to parallel to the optical table and parallel to each diffracted beam. A roof mirror reflecting back the non-diffracted beam in the long-wavelength arm is mounted on a manual translation stage placed on the base plate fixing the roof mirror for the diffracted beam. We can manually adjust the delay of the non-diffracted beam with this setup so as to generate interference fringes with a period appropriate for the range and resolution of a spectrometer. The base plate mounting the two roof mirrors is mounted on another translation stage driven by a piezo actuator such that the displacement of the roof mirror for the diffracted beam caused by the piezo translation stage should be the same as that for the non-diffracted beam. Therefore, the error signal extracted from the interference fringes of the non-diffracted beam can be fed back to the common displacement of both roof mirrors for the two beams through the piezo translation stage. The configuration of the roof mirrors in the short-wavelength arm is almost the same as that in the long-wavelength arm except that the piezo translation stage is replaced by a manual translation stage.

The non-diffracted beams from both arms are spatially superposed on another combiner mirror, and then the superposed non-diffracted beam co-propagates with the diffracted beam (the seed pulse to OPA3) until it passes above the BBO crystal used in OPA3 and is picked up with a folding mirror. We assume that the phase variation of the non-diffracted beam is almost the same as that of the diffracted beam because of the short distances between the separate mirrors used for the diffracted and non-diffracted beams. To determine the stability of the MZI, we can thus monitor the phase variation between short- and long-wavelength components of the non-diffracted beam by measuring the spectral interference fringes appearing near the edge of the reflection wavelength region (~1450 nm) after passing above the BBO crystal. The spectral fringes should be observed if we measure the spectrum of the non-diffracted beam at this stage.

Nevertheless, we did not directly measure the spectrum because the spectral resolution of the commercially available spectrometer used in the SWIR wavelength region is generally insufficient to resolve the fringes. Therefore, we aimed to apply a high-resolution spectrometer in the visible-wavelength region (~480 nm) to resolve the spectral interference fringes. We applied the SFG of the non-diffracted beam by mixing it in a type-II BBO nonlinear crystal with the residual pump beam after OPA3 to up-convert the wavelength to the visible region. The pulse duration of the residual pump beam was increased to approximately 800 fs by inserting a pair of transmission gratings in the beam path because the spectral edge components from the short- and long-wavelength arms should be temporally separated with a time delay of  ~700 fs to generate spectral interference fringes with a period of  ~1 nm, which is appropriate for the high-resolution spectrometer in the visible-wavelength region that we adopted (HR4000, Ocean Insights). By considering the negative chirp given by the grating pair to the residual pump pulse, we adjusted the time order of the two wavelength components so that the shortest edge of the long-wavelength component is advanced to the longest edge of the short-wavelength component. We expect that the broad range of the spectrum in each spectral component will contribute to the generation of the same visible-wavelength component with this time order because the short-wavelength component of the pump pulse temporally overlaps with the long-wavelength component of the non-diffracted beam and vice versa.

In the experiment, we successfully observed clear interference fringes ranging from 476 to 483 nm, as shown in Fig. 4c. The temporal variation of the spectral interference fringes in Fig. 4c was measured under the active feedback control of the path length in the interferometer to stabilize the fringes. We can perform shot-by-shot acquisition (5-ms integration) of each spectrum owing to the high sensitivity to the visible light of the spectrometer, and thus the number of 6 × 10^4^ shots is equivalent to 5 min. The phase extracted from the fringes, as shown in Fig. 4d, exhibits good stability with an rms error of 130 mrad (in-loop). Therefore, we are convinced that the short- and long-spectral components are coherently superposed or synthesized behind the interferometer. The histogram of the relative phase shift is shown in Fig. 4e.

After the MZI setup, the SWIR pulses (diffracted beams) were sent to the OPA3 for amplification.

### OPA3

The pulse duration of an amplified pulse in an OPA should generally be similar to that of the pump pulse to efficiently transfer the energy of the pump pulse to the amplified pulse. To evaluate the evolution of the pulse duration of the amplified pulse in the actual setup in our OPA system, we consider that a Glan–Laser calcite polarizer for the purification of the linear polarization and a window made of CaF_2_ crystal should be used for future applications of the output pulses from the OPA system, such as high-order harmonic generation. This is because the purification of the linear polarization is necessary for the separation of the high-order harmonic beam from the fundamental beam with a high distinction rate when we use a silicon beam splitter mirror under Brewster incidence^26^, and a CaF_2_ plate is conventionally used for the input window of a vacuum chamber. Thus, we inserted a Glan–Laser calcite polarizer with a thickness of 15 mm and a CaF_2_ plate with a thickness of 9.5 mm in the path of the output beam from OPA3. The estimated pulse duration is  ~330 fs in the BBO crystal of OPA3 when we compensate for the dispersion to obtain the TL pulse after passing through these two optics.

We also inserted a pair of transmission gratings with a groove density of 1908 g/mm (PCG-1908-675-972, Ibsen) in the beam path of the pump pulse to adjust the pulse duration. The throughput of the grating pair is 92% and the pulse duration after the optimization of OPA3 is estimated to be 370 fs. The energy of the output pulse from the OPA3 was measured to be 32 μJ with a pump energy of 1.1 mJ and an intensity of  ~30 GW/cm^2^.

After the OPA3, we further performed the spatial and temporal characterization for the amplified SWIR pulses.

### Beam focusing and profile

**Fig. 5:**
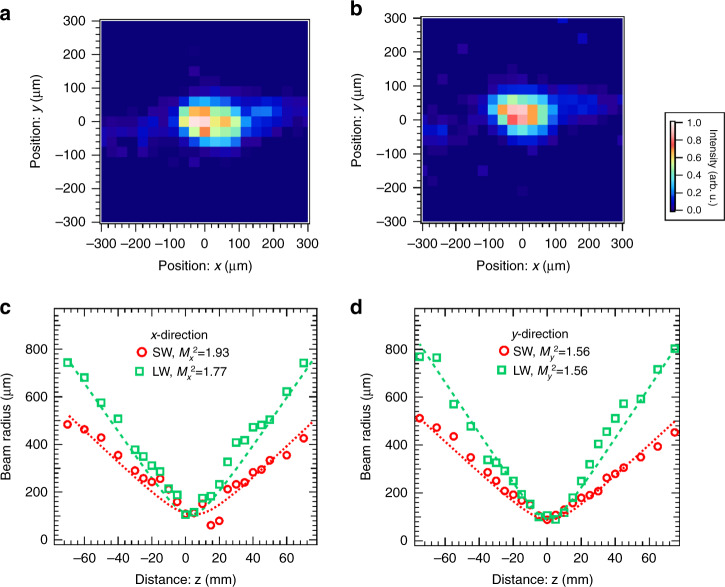
Measurement of the spatial profiles of the output pulses from OPA3. **a** Beam profile of the short-wavelength (SW) component at the focal point. **b** Beam profile of the long-wavelength (LW) component at the focal point. The *x*- and *y*-directions are defined as parallel and perpendicular to the optical table, respectively, in both figures. The offsets of the positions in the *x*- and *y*-directions are common in **a**, **b**. **c** Evolution of the beam radius (half width at e^−2^ maximum) in the *x*-direction with the beam propagation across the focal point. Circles and squares denote the radii of the short- and long-wavelength components, respectively. We evaluated the *M*^2^ value for each wavelength component by fitting each measured radius. Resultant fitting curves are shown as dotted and dashed curves for the short- and long-wavelength components, respectively. **d** Evolution of the beam radius (half width at e^−2^ maximum) in the *y*-direction. Symbols and traces are defined as in **c**.

We measured the spatial characteristics of each spectral component after passing OPA3 to confirm that the two spectral components are spatially superposed. We focused the amplified beam after OPA3 using a concave mirror with a radius of curvature of 800 mm and recorded the beam profile at each position with an IR camera (Xeva-2.35, Xenics) fixed on a translation stage set along the propagation direction of the beam. The beam profiles of the short- and long-wavelength components at the focusing point are shown in Fig. 5a, b, respectively. The beam profile for each wavelength component was individually measured by blocking each arm alternatively in the MZI. The evolutions of the beam radii in the *x*- and *y-* (parallel and perpendicular to the optical table) directions of each spectral component are depicted in Fig. 5c, d, respectively. The curves for the beam radii (in radius at e^−2^ maximum) are obtained by fitting the measured profiles to the Gaussian function. We observe in Fig. 5a, b that the focusing points of both spectral components coincide on the *x*–*y* plane. The focal positions of both spectral components are also the same in the *x*- and *y*-directions, as we can see in Fig. 5c, d. Therefore, we conclude that the wavefronts of the two spectral components are in a good agreement. The *M*^2^ values of the beam profile for the short-wavelength component are evaluated as ${M}_{x}^{2}=1.93$ and ${M}_{y}^{2}=1.56$, and those for the long-wavelength component are evaluated as ${M}_{x}^{2}=1.77$ and ${M}_{y}^{2}=1.56$. A low resolution of camera pixels near the focal point (2–3 pixels/e^−2^ radius) might degrade the *M*^2^ values. The difference in ${M}_{x}^{2}$ values between the short- and long-wavelength components might be attributed to the wavefront difference between the output pulses from two dazzlers in the MZI.

### Pulse characterization

To characterize the pulse duration, we built a 2DSI^20,21^, because the 2DSI technique can be used for characterizing ultrashort pulses in the sub-cycle regime, as was demonstrated by Huang et al.^16,27^. A pair of replica pulses with a spectral shear is generated by SFG with a small part of the pump pulse, then the spectrum of the generated pulse pair is measured with a spectrometer detecting the visible-wavelength region. The details of the setup for the 2DSI measurement are shown in “Methods”.

From the spectrogram of the 2DSI, we retrieved the phase of the generated SWIR pulse and adjusted the pulse shape by manually inputting the obtained parameters into the interface connected to the AOPDFs to change the dispersion conditions of the SWIR pulse. There is no feedback loop between the characterization of the output pulse and the AOPDFs. After several trials, we were able to optimize the dispersion.

**Fig. 6:**
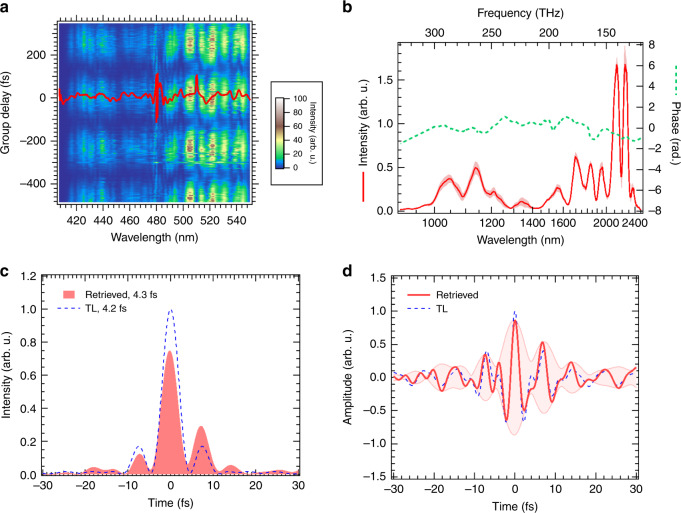
Pulse characterization using two-dimensional shearing interferometer (2DSI). **a** 2DSI spectrogram. The group delay (GD) obtained from the spectrogram is depicted as a solid curve. **b** Measured spectrum (solid curve) of the output pulse from OPA3 and the spectral phase (dashed line) retrieved from the measured GD in **a**. The shaded area indicates the standard deviation for the measured spectra. **c** Temporal profile (intensity) calculated from the measured spectrum and the retrieved phase is shown as a shaded area. The pulse duration is estimated to be 4.3 fs in FWHM, which is comparable to the pulse duration of the transform-limit (TL) pulse, depicted as a dashed curve, with a pulse duration of 4.2 fs. **d** Electric field calculated from the measured spectrum and the retrieved phase is depicted as a solid curve. We assume the CEP to be zero in this calculation. The shaded area specifies the envelope of the electric field. The electric field of the TL pulse is also shown as a dashed curve for comparison.

The two-dimensional spectrogram in Fig. 6a exhibits the spectrum sequence while scanning the phase of one of the replica pulses in the pair. The phase for the vertical axis in this spectrogram is converted to the equivalent delay with the measured frequency shear of 4.1 THz. The solid curve in this figure is the group delay of the measured pulse extracted from the two-dimensional spectrogram.

The phase of the measured pulse is retrieved by integrating the group delay with respect to angular frequency. The resultant phase and measured spectrum of the amplified pulse from OPA3 are shown in Fig. 6b. We evaluated the pulse duration to be 4.3 fs from the temporal profile of the amplified pulse retrieved from the phase and the spectrum shown as a solid curve with a shaded area in Fig. 6c. This is comparable to the duration of the TL pulse of 4.2 fs, depicted as a dashed curve in Fig. 6c. The pulse duration of 4.3 fs is equivalent to 0.73 optical cycles at the carrier wavelength of 1.8 μm determined by the COG frequency of the measured spectrum, and hence, the retrieved optical field with an assumption of zero CEP, depicted as a solid curve in Fig. 6d, exhibits a nearly field-transient-like shape owing to the sub-cycle nature.

### CEP measurement after OPA3

**Fig. 7:**
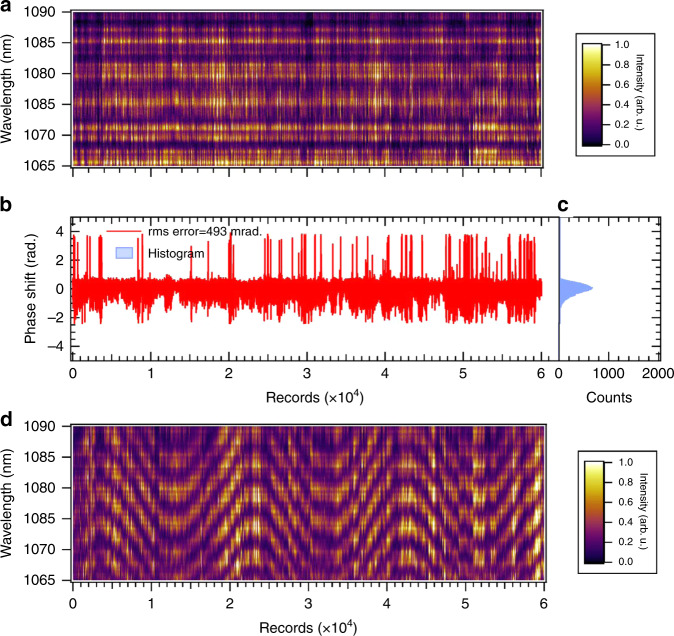
CEP measurement after OPA3. **a** Temporal evolution of the *f*–2*f* spectral interference fringes originating from the output pulse from OPA3 under CEP stabilization control. The acquisition time for each record of the spectrum is equivalent to the repetition period of the laser pulse (5 ms), and thus, the number of records (6 × 10^4^) represents the recording time of 5 min. **b** Relative phase shift (CEP) extracted from the spectrogram depicted in **a**. The rms error of the phase shift is estimated to be 493 mrad. **c** Histogram of the relative phase shift. The width of a bin is 0.01 rad (we set 100 bins/rad) in this histogram. **d** *f*–2*f* spectral interference fringes when we actively modulated the delay between the pump and seed pulses in OPA1 under CEP stabilization control.

We performed the shot-to-shot measurement of the *f*–2*f* interference fringe spectrum for the amplified pulse from OPA3 to determine the CEP stability. The resultant *f*–2*f* interference spectrogram including the 6 × 10^4^ collections of interference spectra and the phase extracted from this spectrogram are shown in Fig. 7a, b, respectively. The histogram of the CEP shift is depicted in Fig. 7c. The rms error of the CEP is estimated to be 493 mrad from the extracted phase under the CEP stabilization with the feedback control loop mentioned in the previous subsection (first and second OPA stages). This phase instability of the amplified pulse is sufficiently low to demonstrate CEP control via the feedback control loop in OPA1 and OPA2. We present a demonstration of the CEP control of an amplified pulse with a sinusoidal shape by scanning the CEP offset in the feedback control loop shown as an *f*–2*f* interference spectrogram in Fig. 7d.

## Discussion

The energy extraction efficiency in OPA3 (2.9%) is relatively low compared with those reported in previous studies^12^. This is because of the degradation of the spatial quality of the pump pulse. The beam profile of the pump pulse exhibited a ellipsoidal shape resulting from the astigmatism accumulated with many reflections from 45° folding mirrors, the surfaces of which were typically convex owing to the tension of the dielectric film stack coating. In addition, spatial clippings of the beam profile due to the limited size of the folding mirrors further degraded the pump beam quality. We will improve the beam quality by inserting a spatial filter, as was adopted in the previous studies^12^, in the beam path of the pump pulse when we add an OPA stage in the future. Currently, we prioritize the simplicity of the pumping beam path.

Another issue is the decrease in the CEP stability measured behind OPA3 compared with that measured behind OPA2. We suppose that this decrease in the stability emerges from the intensity fluctuation of the amplified pulse in OPA3 owing to the coupling effect of the intensity and the CEP. In general, the coupling effect is explained in a context of WLG in an *f*–2*f* interferometer^22,28^; thus, we can expect the actual CEP fluctuation to be less than the measured fluctuation. Nevertheless, we did not generate white-light pulses for the CEP measurement in this study, and hence we must disregard this effect in the *f*–2*f* interferometer. We suppose, instead, that the coupling effect is due to the dispersions of the nonlinear refractive indices of the dispersion compensation plates of the calcite polarizer, CaF_2_ window at the exit of OPA3, and an s-TIH6 glass (OHARA Inc.) plate placed in front of the *f*–2*f* interferometer for adjusting the period of spectral fringes. We describe our model to explain how the intensity fluctuation *δI* is transferred to the CEP fluctuation *δϕ*_CEP_ in the subsection “Estimation of CEP fluctuation” in “Methods”.

As a result, we estimated that the *δϕ*_CEP_s originating from the calcite polarizer with a thickness of 15 mm, the CaF_2_ plate with a thickness of 9.5 mm, and the s-TIH6 plate with a thickness of 14 mm were 172–285, 3.0–4.7, and 138–259 mrad, respectively. This estimation reasonably explains the CEP degradation.

We expect the CEP stability to be improved by installing the spatial filter mentioned above, because the fluctuation of the pulse energy is mainly caused by the shot-to-shot pointing instability of the pump pulse with the shot-to-shot fluctuation of air flow and/or the nonlinear phase shift acquired in a long beam path in air. The use of a vacuum tube with the spatial filter is beneficial for eliminating these effects.

The up-conversion of the SWIR spectrum with interference fringes to the visible-wavelength region, which is executed for the active stabilization of the interferometer, is a trivial idea but generally useful for detecting the relative phase difference between the ultrashort pulses in the IR region without using an IR spectrometer containing an expensive and high-dark-current IR detector.

The control ranges for the dispersions in both AOPDFs are still sufficient to compensate for additional dispersions induced by multiple transmitting materials with thicknesses of mm order. This situation is beneficial for pulse energy scaling since it is only necessary to install additional OPA stages with more energetic pumping sources behind OPA3 without additional devices for dispersion management such as chirped mirrors. Therefore, we expect the amplification of sub-cycle pulses up to 1 mJ order by adding an OPA stage^29^ pumped by a Ti:sapphire laser pulse with an energy of 10 mJ order^23^ in the future after improving the pump beam quality and also the CEP stability in the OPA3.

In summary, we demonstrated the amplification of sub-cycle SWIR pulses in a cascaded OPA chain with features of (i) a gain bandwidth of over one octave in a BBO crystal pumped by a “red” (708 nm) pulse and (ii) the division and synthesis of the wavelength components to control the dispersion with two AOPDFs before OPA3. The former feature has the advantage of a short pulse duration in the sub-cycle regime compared with the OPA chains, which consist of an OPA chain using BIBO crystals pumped by conventional Ti:sapphire laser pulses, as reported in previous studies^12^. Regarding the latter feature, the conventional MZI used for the division and synthesis is simple and robust against factors reducing stability, in contrast to the waveform synthesis of amplified pulses after passing through m-scale optical paths in multiple OPA chains with different-color pump sources, even though the interferometer introduces some complexity into the OPA chain and the gain bandwidth of one OPA chain with a single-color pump source cannot be extended from the IR to UV regions, as was achieved with a genuine waveform synthesizer.

## Methods

### White-light generation

Part of the pump pulse with an energy of  ~20 μJ was sent to the beam line for WLG as a seed pulse for OPA1. A crystal plate of calcium fluoride (CaF_2_) with a thickness of 3 mm was placed at the focus of a one-to-one telescope composed of two concave mirrors with an identical radius of curvature of 200 mm. We can finely adjust the beam diameter and the energy of the input pulse with an iris and a variable attenuator set in front of the telescope. Note that we examined three types of crystal as a nonlinear material for WLG before selecting the CaF_2_ plate. The candidate materials other than CaF_2_ were yttrium aluminum garnet (YAG) and sapphire^30^, which were considered on the basis that the nonlinear indices of both materials are higher than that of CaF_2_. We summarize the nonlinear refractive indices^31^ of these materials in Table 1. Critical powers, *P*_cr_,^32^ estimated at a wavelength of 708 nm for self-focusing and filamentation are also shown in Table 1.

**Table 1:** Parameters of crystals used for white-light generation (WLG).

Material	*n*_2_ (cm^2^/W)	*P*_cr_ (MW)
CaF_2_	1.24 × 10^−16^	4.3
Sapphire	3.1 × 10^−16^	1.4
YAG	6.9 × 10^−16^	0.6

*n*_2_ is the nonlinear refractive index and *P*_cr_ is the critical power for WLG.

We had expected that a sapphire or YAG plate might efficiently generate the white light owing to a high nonlinear refractive index and low critical power, while the low dispersion of a CaF_2_ plate might be beneficial for broadening the white-light spectra^33^.

**Fig. 8:**
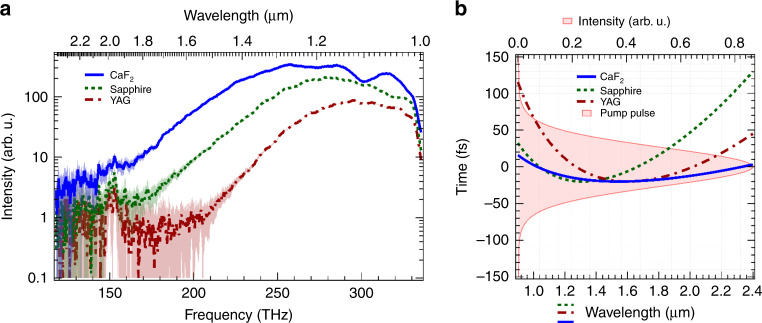
White-light generation. **a** White-light spectra generated from a 3-mm CaF_2_ (blue solid curve), a 3-mm sapphire (green dotted curve), and a 3-mm YAG (brown one dot chain curve). The shaded area are error bars estimated from the statistics of the measured spectra and backgrounds. **b** Calculated group delay introduced by the CaF_2_ (blue solid curve), the sapphire (green dotted curve), and the YAG (brown one dot chain curve). The pump pulse profile calculated under the Fourier limit condition is depicted with the shaded area.

The measured spectra of the white-light generated from CaF_2_, YAG, and sapphire plates are shown as blue solid, green dotted, and brown dash-dot curves in Fig. 8a, respectively. The thicknesses of these plates are all 3 mm. We adjusted the input pulse energy for each plate to be as high as possible using a variable attenuator and an aperture under the upper limit energy to reveal a rapid oscillating structure on the white-light spectrum, which was known as a result of the refocusing of the filament^34^, in the relevant wavelength range of the SWIR (from approximately 1–2.4 μm). The resultant pulse energies were 2.9 μJ for the CaF_2_ plate, 1.3 μJ for the sapphire plate, and 0.65 μJ for the YAG plate.

We found that the intensity of the white-light spectrum generated from the CaF_2_ plate was the highest in this figure. The wavelength of the spectrum was extended to 2.4 μm. This result is due to the highest available input pulse energy and the lowest dispersion for the CaF_2_ plate. As for the dispersion, the difference between the maximum and minimum group delays for the SWIR region in the CaF_2_ plate was evaluated to be less than 40 fs, shown as a blue solid curve in Fig. 8b, which is only less than 30% of that in the sapphire and YAG plates, shown as a green dotted and a brown dash-dot curve in Fig. 8b, respectively. The group delay of less than 40 fs ensured the temporal overlap of the pump pulse with a duration of 60 fs, shown as a shaded area in Fig. 8b, to the whole spectrum in the SWIR region. Therefore, we adopted the CaF_2_ plate for the WLG in the OPA system.

The highest pulse energy of the input pulse to the CaF_2_ plate comes at the cost of the need to compensate for the lowest nonlinear refractive index, and thus, it leads degradation of the plate in a long term use. We observed the instability of the spectrum of the idler pulse after  ~20-min WLG and damage appeared in the plate^33,35^. To solve this problem, the CaF_2_ plate was continuously moved perpendicular to the beam path. A two-axis translation stage driven by piezo motors is used for the two-dimensional control of the position of the CaF_2_ plate to track a Lissajous-curve-like trajectory. The speed of the stage was adjusted to prevent the degradation of the CaF_2_ plate; it should be sufficiently low nor to cause the instability of the resulting pulses. After some trial and error between these tradeoffs, we set the speed of the stage to  ~50 μm/s. The CEP measurements were performed with this CaF_2_ plate movement.

### Octave-spanning bandwidth via degenerate OPA

To obtain a broad bandwidth in the OPA, the phase mismatch Δ*k* = *k*_s_ + *k*_i_ − *k*_p_ should be nearly zero in the broad spectral range, where *k*_s_, *k*_i_, and *k*_p_ are the wavenumbers for the signal, idler, and pump, respectively. The Taylor expansion of the phase (wavenumber) mismatch Δ*k* is^36^

1 $$\Delta k={k}_{\text{p}}({\omega }_{\text{po}})-[{k}_{\text{s}}({\omega }_{\text{so}}+\Delta {\omega }_{\text{s}})+{k}_{\text{i}}({\omega }_{\text{io}}+\Delta {\omega }_{\text{i}})]$$

2 $$=\,	{k}_{\text{p}}({\omega }_{\text{po}})-[{k}_{\text{s}}({\omega }_{\text{so}})+{k}_{\text{i}}({\omega }_{\text{io}})]\\ 	-\mathop{\sum }\nolimits_{n = 1}^{\infty }\frac{1}{n!}\left[{\left.\frac{{\partial }^{n}{k}_{\text{s}}}{\partial {\omega }_{\text{s}}^{n}}\right|}_{{\omega }_{\text{s}} = {\omega }_{\text{so}}}\Delta {\omega }_{\text{s}\,}^{n}\ +{\left.\frac{{\partial }^{n}{k}_{\text{i}}}{\partial {\omega }_{\text{i}}^{n}}\right|}_{{\omega }_{\text{i}} = {\omega }_{\text{io}}}\Delta {\omega }_{\text{i}\,}^{n}\right],$$

where Δ*ω*_s_ = *ω*_s_ − *ω*_so_ and Δ*ω*_i_ = *ω*_i_ − *ω*_io_ specify the offsets from the central angular frequency for the signal and idler waves, respectively. The expansion was obtained by assuming a monochromatic pump source. For the fixed pump angular frequency, *Δω*_s_ is equal to  −Δ*ω*_i_. On the other hand, under the angle phase-matching condition, the phase-matching criterion can be obtained at a particular wavelength to make Δ*k*_o_ ≡ *k*_p_(*ω*_po_) − [*k*_s_(*ω*_so_) + *k*_i_(*ω*_io_)] = 0 in Eq. (1). Under the conditions of signal-idler degeneracy and a fixed pump frequency, all the odd terms thus vanish, i.e.,

3 $${\left.\frac{{\partial }^{2n+1}{k}_{\text{s}}}{\partial {\omega }_{\text{s}}^{2n+1}}\right|}_{{\omega }_{\text{s}} = {\omega }_{\text{o}}}-\ \ {\left.\frac{{\partial }^{2n+1}{k}_{\text{i}}}{\partial {\omega }_{\text{i}}^{2n+1}}\right|}_{{\omega }_{\text{i}} = {\omega }_{\text{o}}}=0.$$

In this case, the second-order derivative (GDD) is crucial for broadening the bandwidth of the phase matching. The GDD can be eliminated by tuning an appropriate pump wavelength for the nonlinear crystal. For a BBO crystal, the zero GDD occurs around a pump wavelength of  ~700 nm. To meet this condition, we thus tuned the central wavelength of a Ti:sapphire laser from 800 to  ~708 nm, as reported in the previous section.

We note that the above-mentioned analysis of the phase-mismatch function is based on the Taylor expansion of Δ*k* in terms of the degenerate angular frequency. On the other hand, we numerically found that the slight detuning of the angle of the optical axis, *θ*, realizes the phase matching at multiple angular frequencies (or wavelengths). This is the reason why the gain spectrum shown in Fig. 2b is broadened and modulated with a slight increase in *θ*.

We calculated the parametric gain for a BBO crystal of length *L* using^37,38^

4 $$G={\text{cosh}}^{2}(gL)+{\left(\frac{\Delta k}{2g}\right)}^{2}{\text{sinh}}^{2}(gL),$$

where we define *g* and Γ as

5 $$g\equiv \sqrt{{\Gamma }^{2}-\frac{\Delta {k}^{2}}{4}},$$

6 $${\Gamma }^{2}\equiv \frac{{\omega }_{\text{s}}\ {\omega }_{\text{i}}}{{n}_{\text{s}}\ {n}_{\text{i}}}\frac{{\chi }_{\text{eff}}^{2}}{{c}_{0}^{2}}{E}_{\text{p}\,}^{2}.$$

The refractive indices for the signal, idler, and pump are denoted as *n*_i_, *n*_s_, and *n*_p_, respectively, in Eqs. (5) and (6), and the following equations. *χ*_eff_ is the effective nonlinear susceptibility, *c*_0_ is the light velocity, and *E*_p_ is the electric field of the pump beam, which is related to the pump intensity *I*_p_ as ${I}_{\text{p}}={n}_{\text{p}}{c}_{0}{\varepsilon }_{0}{E}_{\text{p}\,}^{2}/2$, where *ε*_0_ is the permittivity of vacuum. The two-dimensional spectrogram for the gain profile depicted in Fig. 2a was calculated from Eqs. (4)–(6), substituting the model parameters of *L* = 3 mm, *χ*_eff_ = 1.8 pm/V, and *I*_p_ = 60 GW/cm^2^ under the degenerate condition.

### *f* –2*f* interferometer for CEP measurement

We built two sets of *f*–2*f* interferometers^39^. One is used for the measurement and control of the CEP of the output pulse from OPA2. The other is used for the measurement of the CEP of the output pulse from OPA3. The structures of these *f*–2*f* interferometers are identical and conventional. Each interferometer is composed of a front aperture, a focusing lens, a BBO crystal for second-harmonic generation, a collimation lens, a polarizer, another focusing lens to the fiber input, and a fiber connector. The WLG setup is not required in the interferometer because the spectral width of the OPA output is broader than one octave. All the optical components were aligned in a commercial 16-mm cage system (Thorlabs Inc.) such that the footprint of the interferometer is as small as possible. We adjusted the period of *f*–2*f* interference fringes by inserting a dispersion plate made of s-TIH6 (OHARA) glass with a thickness of 14 mm set in front of the interferometer to clearly resolve the fringes.

The part of the output pulse from OPA2 sent to the *f*–2*f* interferometer was picked up via the Fresnel reflection from the output surface of the BBO crystal in OPA2. In this case, it is possible to minimize the loss of the pulse energy sent to the next stage by omitting the beam splitter used for the CEP measurement, although the intensity of the picked up pulse is not sufficiently high to detect the *f*–2*f* spectral signal with a low-cost CCD array. Therefore, we used a spectrometer (Maya2000Pro, Ocean Insight) with a high-sensitivity detector (S110, Hamamatsu), although a high-sensitivity detector usually requires a long integration time. Actually, the minimum integration time of the spectrometer is longer than 10 ms (two periods of pulse repetition). This is why we recorded the three-shot average of the spectral fringes for the CEP measurement of the OPA2 output.

In contrast, a conventional high-resolution spectrometer (HR4000, Ocean Insight) can be used to measure the *f*–2*f* interference fringes of intense output pulses from OPA3. Thus, we were able to perform the shot–to–shot measurement to obtain the *f*–2*f* interference spectrogram shown in Fig. 7a.

### Estimation of CEP fluctuation

We propose a candidate model to explain the CEP fluctuation in this subsection. A CEP change of a pulse, *ϕ*_CEP_, after propagating in a medium with a length of *L* is defined as the difference between the carrier phase of *kL* and the group phase of *ωτ* as

7 $${\phi }_{{\rm{CEP}}}=kL-\omega \tau =2\pi L\frac{dn}{d\lambda },$$

where *k* is the wavenumber, *ω* is the angular frequency, and *τ* is the group delay defined as

8 $$\tau =L\frac{dk}{d\omega }.$$

The second equality in Eq. (7) is obtained by putting *k* = *n* ⋅ 2*π*/*λ*, and *ω* = 2*πc*/*λ* in Eq. (7) and by using Eq. (8), where *n*, *c*, and *λ* are the refractive index, the velocity of light in vacuum, and the wavelength, respectively. We assume that the refractive index *n* is expressed as *n*_0_ + *n*_2_*I*, namely, it is the summation of a refractive index *n*_0_ independent of the laser intensity *I* and a refractive index proportional to *I* with a coefficient of the nonlinear refractive index *n*_2_. We suppose that *ϕ*_CEP_ shifts to *ϕ*_CEP_ + *δϕ*_CEP_ when the laser intensity deviates from *I* to *I* + *δI*. Finally, we obtain the relation between *δϕ*_CEP_ and *δI*,

9 $$\delta {\phi }_{{\rm{CEP}}}=2\pi L\frac{d{n}_{2}}{d\lambda }\delta I,$$

by substituting *n* = *n*_0_ + *n*_2_*I* in Eq. (7). Therefore, we can estimate the *δϕ*_CEP_ caused by the intensity fluctuation of *δI* with knowledge of the dispersion of the nonlinear refractive index *dn*_2_/*dλ*.

We evaluated the dispersions of the nonlinear refractive index of the materials related to the CEP measurement in accordance with a model reported in ref. ^40^. The band-gap energy of a material is essential for the determination of *dn*_2_/*dλ* in this model. The magnitude of *dn*_2_/*dλ* for the extraordinary ray in calcite was estimated to be 2.2–3.7 × 10^−14^ (m^2^/W)/m in the wavelength region of 1.4–1.7 μm using Eq. (7) in ref. ^40^ with a band-gap energy of 5.07 eV^41^. We also estimated the ∣*dn*_2_/*dλ*∣ of s-TIH6 glass to be 1.7–3.2 × 10^−13^ (m^2^/W)/m in the same wavelength region by using the band-gap energy of 3.31 eV determined from the Tauc plot^42^ with its absorption coefficient of this material^43^. A large band-gap energy of CaF_2_ (9.92 eV^44^) compared with those of the other two materials was responsible for the reduction in ∣*dn*_2_/*dλ*∣ down to 6.1–9.8 × 10^−16^ (m^2^/W)/m.

The intensity fluctuations *δI* at the entrance of the calcite polarizer and the CaF_2_ plates were evaluated to be 8 GW/cm^2^ and that of the s-TIH6 plate was 0.9 GW/cm^2^ from the measured fluctuation (standard deviation) of the pulse energy. The length of each material was already described in “Discussion”. We obtained *δϕ*_CEP_s mentioned in “Discussion” by putting the numerical values evaluated in this subsection into Eq. (9).

### 2DSI measurement

To characterize the group delay of an ultrashort optical pulse by 2DSI measurement, we must generate two replicas of the measured ultrashort optical pulse with a frequency shear and without temporal delay^20,21^. We generated the two replica pulses by the SFG of the measured pulse mixed with the pump pulse in a manner similar to that reported in refs. ^45,46^. Part of the pump pulse was picked up with a 5% beam splitter mirror set in front of OPA3 and sent to a 4–*f* zero–dispersion stretcher consisting of a reflective grating, an achromatic lens, and folding mirrors. The grating was configured on a Fourier plane of the achromatic lens and the two folding mirrors aligned side-by-side are set on another Fourier plane. The pump pulse was diffracted with the grating to be angularly dispersed and then collimated with the achromatic lens. The pulse was divided into short and long spectral components by the reflection from two folding mirrors owing to the spatial dispersion of the collimated pulse. We placed a double slit in front of the two folding mirrors to reduce the spectral width of each component. The relative phase *ϕ* between the two spectral components was controlled with a piezo stage supporting one of the folding mirrors. The spatial dispersion was removed by the second diffraction of the pulse focused with the achromatic lens.

The pulse was then sent to the SFG stage including a BBO crystal with a thickness of 0.1 mm for the type-II phase-matching condition with the measured pulse. The acceptable bandwidth in the SFG process is sufficiently large to convert the whole SWIR spectrum of the measured pulse to the visible region. The shear frequency Δ*ν* was set to 4.1 THz, which was calibrated by measuring the wavelength shift of the visible spectrum of one replica pulse generated with the pump pulse passing through one slit relative to the spectrum of another replica pulse. We recorded spectra of the visible SFG pulses by scanning *ϕ* to obtain the 2DSI spectrogram shown in Fig. 6a. The relative phase was converted to the group delay *τ* using *τ* = *ϕ*/(2*πΔν*) in this spectrogram.

We examined the accuracy of our 2DSI measurement before implementing the dispersion compensation in the entire SWIR spectrum. This is reported in Supplementary Information. Please refer to Figs. S1 and S2 in the Supplementary Information.

We note that a coherent artifact in the 2DSI measurement, which was studied by Rhodes and coworkers^47^, can be excluded from our measurement. The coherent artifact originates from the shot-to-shot instability of the spectral phase of the measured pulse sequence and from the averaged data recordings of multiple pulses accompanied by this instability. In our study, we performed the shot-to-shot measurement of the *f*–2*f* spectral interference fringes as shown in Fig. 7a–d. When phase fluctuation such as thermal noise adopted in the simulation studies in ref. ^47^ was present in the measured pulse, we were not able to stabilize or control either the shot-to-shot *f*–2*f* spectral interference fringes shown in these figures. We confirmed that the fluctuation of the pulse duration was approximately 3% by statistical simulations, in which we added the random phase fluctuation or random GDD fluctuation to the measured phase so as to reproduce the measured noise level (standard deviation) of the CEP (493 mrad), supposing that the measured noise might be regarded as a phase noise other than CEP noise.

## Supplementary information

Supplementary Information

## Data Availability

The data that support the findings of this study are available from the corresponding author upon request.
