# Supplementary material for: Optical parametric amplification of sub-cycle shortwave infrared pulses
Source: Nat Commun. 2020 Jul 8;11:3413. doi: 10.1038/s41467-020-17247-9 (PMC7343886; doi:10.1038/s41467-020-17247-9)
Supplement: Supplementary file 1 — Supplementary Information [file 41467_2020_17247_MOESM1_ESM.pdf]

Optical parametric amplification of sub–cycle shortwave infrared pulses Supplementary Information

Yu-Chieh Lin,[1,*] Yasuo Nabekawa,[1] and Katsumi Midorikawa[1]

[1]*Attosecond Science Research Team,*
Extreme Photonics Research Group,
RIKEN Center for Advanced Photonics,
2-1 Hirosawa, Wako, Saitama 351-0198, Japan

[*] yu-chieh.lin@riken.jp

GDD control revealed by 2DSI measurements

Before implementing compensation for dispersion of the amplified pulse, we examined the accuracy of the two–dimensional shearing interferometry (2DSI) measurement by applying fine group–delay–dispersion (GDD) changes using an acousto–optic programmable dispersive filter (AOPDF) set in front of OPA3. We show 2DSI spectrograms measured with different GDD values in Figs. **S1**(a)–(d). Other high–order dispersions (3rd– and 4th–order dispersions can be controlled with the AOPDF) were fixed in these measurements. For the sake of simplicity, we only measured the long–wavelength component amplified with OPA3 by blocking the short–wavelength component. The vertical axes in the spectrograms were converted to the equivalent delay with the measured frequency shear of 2.9 THz. The phase in each measurement was retrieved by integrating each group delay (GD) extracted from each 2DSI spectrogram with respect to angular frequency. The GDD values applied to the pulse with the AOPDF are –2010 fs^2 , –1970 fs^2, –1980 fs^2, and –1990 fs^2 to obtain the spectrograms shown in Figs. **S1**(a), (b), (c), and (d), respectively. These values are defined at the wavelength of 2000 nm.

We obtained the phase differences between the two of four phases retrieved from the measured 2DSI spectrograms, as shown in Fig. **S2**(a). The solid curves are the phase differences retrieved from the 2DSI spectrograms, and the dashed curves show the phase differences applied to the pulses with the AOPDF. The curves labeled with ΔGDD=40 fs^2, ΔGDD=30 fs^2, ΔGDD=20 fs^2, and ΔGDD=10 fs^2 represent the phase differences between the phases retrieved from the 2DSI spectrograms in Figs. **S1**(a) and (b), Figs. **S1**(a) and (c), Figs. **S1**(a) and (d), and Figs. **S1**(c) and (d), respectively.

The applied phase differences depicted as dashed curves in Fig. **S2**(a) agree well with the retrieved phase differences depicted as solid curves. The deviation of each measured phase difference from each applied phase difference is shown in Fig. **S2**(b). The root mean square value of the deviation evaluated from the four traces in Fig. **S2**(b) is 273 mrad., which is sufficiently low to accurately compensate for the dispersion of the measured pulse to obtain the near–Fourier–limit pulse. We note that the frequency shear of 4.1 THz in the 2DSI measurement presented in the main article is 1.4 times larger than 2.9 THz. The GD resolution with the 4.1–THz shear should be 1.4 times finer than that with the 2.9–THz shear.

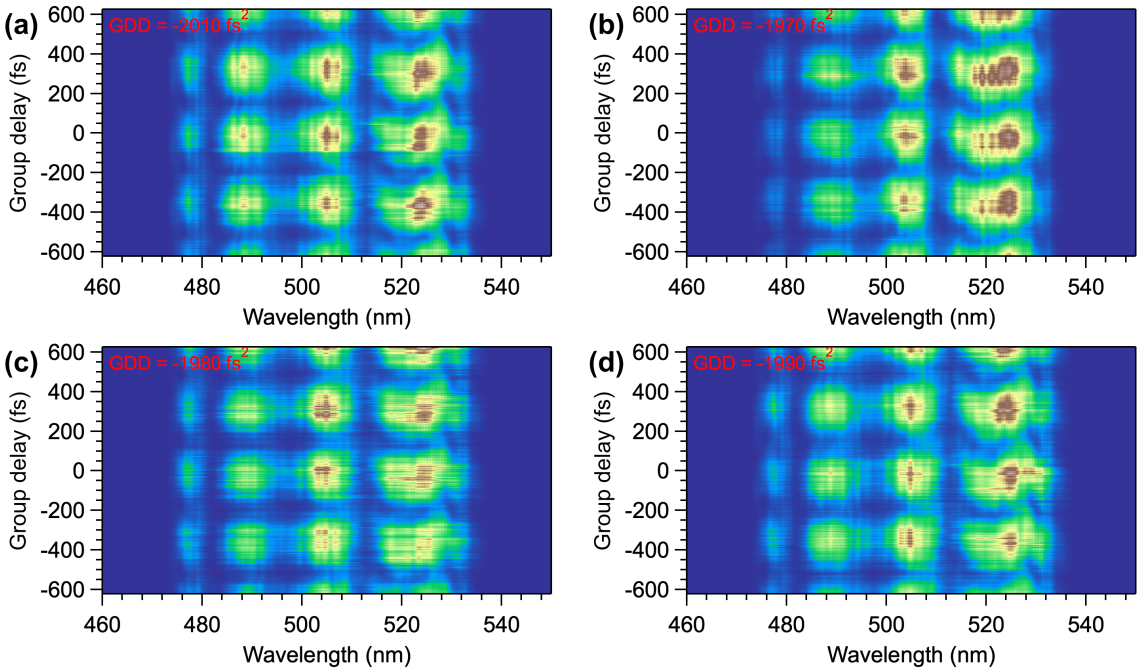

Fig. S1. Two–dimensional shearing interferometer (2DSI) spectrograms measured with fine group–delay–dipsersion (GDD) changes. 2DSI spectrograms obtained when applying a GDD of –2010 fs^2: (a), –1970 fs^2: (b), –1980 fs^2: (c), and –1990 fs^2: (d). GDD was applied to the measured pulses by using an acousto–optic programmable dispersive filter (AOPDF).

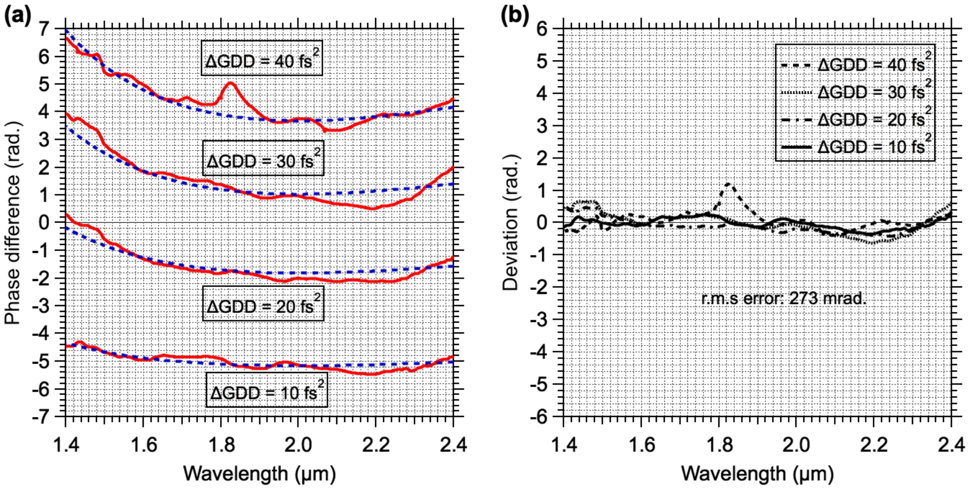

Fig. S2. Phase differences retrieved from 2DSI spectrograms. (a) Phase differences retrieved (solid curves) and applied (dashed curves). Applied GDD difference values (ΔGDD) are indicated with labels put close to each pair of solid and dashed curves. (b) Deviation of the retrieved phase difference from the applied phase difference for each ΔGDD.
